# Terbium(III) hydrogendiphosphate(V) tetra­hydrate

**DOI:** 10.1107/S1600536808001992

**Published:** 2008-01-23

**Authors:** Karla Fejfarová, Rachid Essehli, Brahim El Bali, Michal Dušek

**Affiliations:** a Institute of Physics, Na Slovance 2, 182 21 Praha 8, Czech Republic; b Department of Chemistry, Faculty of Sciences, University Mohammed 1st, PO Box 524, 60 000 Oujda, Morocco

## Abstract

The Tb atom of the title compound, TbHP_2_O_7_·4H_2_O, is coordinated by the O atoms of three symmetrically independent water mol­ecules and by five O atoms belonging to HP_2_O_7_^−^ groups. The TbO_8_ polyhedra are inter­connected by the diphospate anions, forming a three-dimensional network which is additionally stabilized by O—H⋯O hydrogen bonding between water mol­ecules and O atoms of the HP_2_O_7_^−^ anions. Uncoordinated water mol­ecules are situated in channels and are connected *via* hydrogen bonds with the framework.

## Related literature

Isostructural compounds of the type REHP_2_O_7_·4H_2_O were reported for RE = Sm by Chehimi-Moumen *et al.* (2002 ▶), for RE = Gd by Hraiech *et al.* (2005 ▶) and for RE = Eu by Anna-Rabah *et al.* (2006 ▶).

## Experimental

### 

#### Crystal data

TbHP_2_O_7_·4H_2_O
*M* *_r_* = 405.9
Monoclinic, 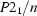

*a* = 6.6006 (6) Å
*b* = 11.4744 (9) Å
*c* = 11.7252 (13) Å
β = 92.150 (8)°
*V* = 887.42 (15) Å^3^
*Z* = 4
Mo *K*α radiation
μ = 8.38 mm^−1^
*T* = 295 K
0.14 × 0.06 × 0.03 mm

#### Data collection

Oxford Diffraction Xcalibur 2 diffractometer with Sapphire 2 CCD area-detector
Absorption correction: analytical [*CrysAlis RED* (Oxford Diffraction, 2005 ▶), using a multifaceted crystal model based on expressions derived by Clark & Reid (1995 ▶)] *T*_min_ = 0.329, *T*_max_ = 0.635
8671 measured reflections
1850 independent reflections
1628 reflections with *I* > 3σ(*I*)
*R*_int_ = 0.019

#### Refinement

*R*[*F* ^2^ > 2σ(*F* ^2^)] = 0.011
*wR*(*F* ^2^) = 0.034
*S* = 1.21
1850 reflections
155 parameters
10 restraints
H atoms treated by a mixture of independent and constrained refinement
Δρ_max_ = 0.29 e Å^−3^
Δρ_min_ = −0.21 e Å^−3^

### 

Data collection: *CrysAlis CCD* (Oxford Diffraction, 2005 ▶); cell refinement: *CrysAlis RED* (Oxford Diffraction, 2005 ▶); data reduction: *CrysAlis RED*; program(s) used to solve structure: *SIR2002* (Burla *et al.*, 2003 ▶); program(s) used to refine structure: *JANA2000* (Petříček *et al.*, 2007 ▶); molecular graphics: *DIAMOND* (Brandenburg & Putz, 2005 ▶); software used to prepare material for publication: *JANA2000*.

## Supplementary Material

Crystal structure: contains datablocks global, I. DOI: 10.1107/S1600536808001992/wm2172sup1.cif

Structure factors: contains datablocks I. DOI: 10.1107/S1600536808001992/wm2172Isup2.hkl

Additional supplementary materials:  crystallographic information; 3D view; checkCIF report

## Figures and Tables

**Table 1:** Selected bond lengths (Å)

Tb1—O1	2.3145 (17)
Tb1—O2^i^	2.2877 (17)
Tb1—O3^ii^	2.3514 (18)
Tb1—O5	2.3718 (18)
Tb1—O6^iii^	2.3842 (17)
Tb1—O8	2.605 (2)
Tb1—O9	2.433 (2)
Tb1—O10	2.421 (2)
P1—O1	1.5129 (18)
P1—O2	1.5063 (18)
P1—O3	1.5169 (19)
P1—O7	1.6201 (18)
P2—O4	1.562 (2)
P2—O5	1.4957 (19)
P2—O6	1.4891 (18)
P2—O7	1.6116 (18)

Symmetry codes: (i) 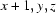; (ii) 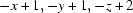; (iii) 
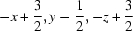
.

**Table 2:** Hydrogen-bond geometry (Å, °)

*D*—H⋯*A*	*D*—H	H⋯*A*	*D*⋯*A*	*D*—H⋯*A*
O4—H41⋯O11	0.813 (13)	1.736 (14)	2.546 (3)	174 (3)
O8—H81⋯O1^ii^	0.82 (2)	1.98 (3)	2.762 (3)	159 (3)
O8—H82⋯O3^i^	0.822 (18)	2.231 (14)	2.972 (3)	150 (3)
O9—H91⋯O4^iv^	0.83 (3)	2.06 (3)	2.851 (3)	161 (3)
O9—H92⋯O8^iii^	0.828 (18)	1.95 (2)	2.750 (3)	164 (3)
O10—H101⋯O5^iii^	0.82 (2)	2.16 (3)	2.880 (3)	147 (3)
O10—H102⋯O7^v^	0.812 (16)	2.28 (2)	2.991 (3)	147 (3)
O11—H111⋯O3^v^	0.80 (2)	2.18 (2)	2.941 (3)	157 (3)

Symmetry codes: (i) 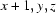; (ii) 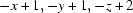; (iii) 
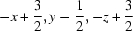
; (iv) 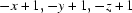; (v) 
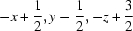
.

**Fig. 1.**
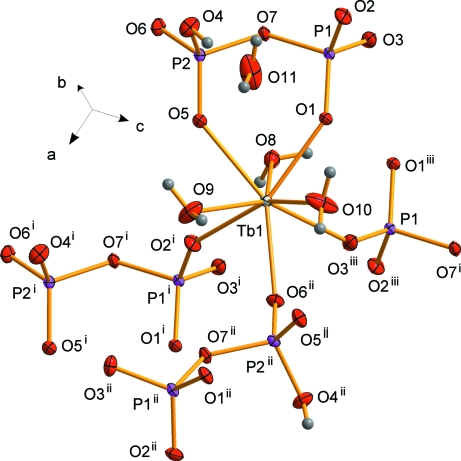
Part of the structure of HTbP2O7.4H2O drawn with displacement ellipsoids at the 50% probability level. [Symmetry codes: (i) 1 + x, y, z; (ii) 1.5 - x, -1/2 + y, 1.5 - z; (iii) 1 - x, 1 - y, 2 - z]

**Fig. 2.**
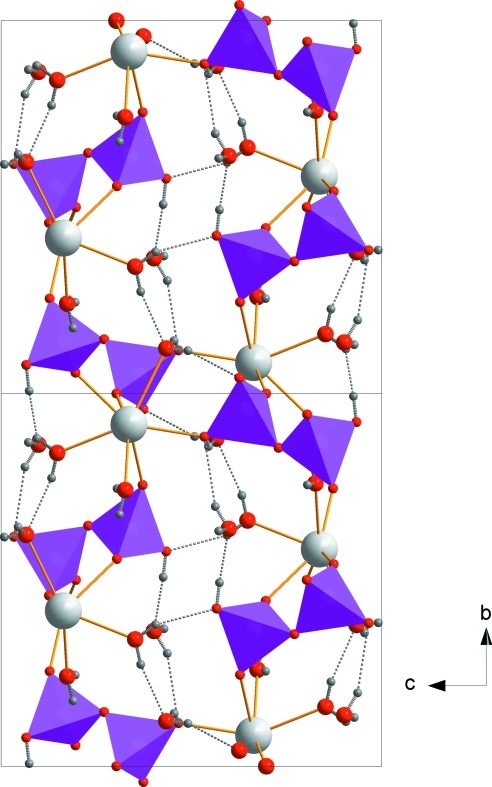
The packing of TbHP2O7.4H2O viewed along a. Hydrogen bonds are represented by dashed lines. Colour code: Pink (P2O7 polyhedra), red spheres (O), grey spheres (Tb), dark grey spheres (H). All atoms are displayed with arbitrary radii. For clarity, O atoms belonging to PO4 tetrahedra have a smaller size than O atoms of water molecules. O atoms that would obscure H atoms important for understanding the hydrogen bonding scheme are plotted semitransparently.

## supplementary crystallographic information

### Comment

Acidic rare earth diphosphates of general formula REHP_2_O_7_^.^nH_2_O exhibit interesting luminescent and optical properties (Hraiech *et al.*, 2005 and references herein). The title compound is isostructural with other compounds of formula type REHP_2_O_7_^.^4H_2_O, RE = Sm (Chehimi-Moumen *et al.*, 2002), Gd (Hraiech *et al.*, 2005), and Eu (Anna-Rabah *et al.*, 2006).

The structure of TbHP_2_O_7_^.^4H_2_O is made up of TbO_8_ polyhedra and HP_2_O_7_ groups that form a three-dimensional framework. In channels running along *a* (Fig. 2) free water molecules are located which are connected *via* hydrogen bonds with the framework (see hydrogen-bonding Table).

The P_2_O_7_ group is protonated, with the H atom located at O4 (Fig. 1), as also indicated by elongation of the corresponding P—O distance. The bridging angle P1—O7—P2 between the two PO_4_ tetrahedra is 130.73 (11)°.

### Experimental

An aqueous solution of TbCl_3_^.^6H_2_O (0.1*M*) was added dropwise to anhydrous Na_4_P_2_O_7_ dissolved in destilled water (0.1*M*). The pH of the mixture was controlled with diluted hydrochloric acid to be slightly acidic, and the solution was stirred for two h at room temperature. Prismatic-shaped colourless crystals with a maximal size of 0.3 mm formed after a few days on slow evaporation.

### Refinement

The H atoms were localized from a difference Fourier map. Their coordinates were refined independently with O—H distances restrained to 0.82 (1) Å. The isotropic temperature parameters of the H atoms were refined with 1.2*U*_eq_ of the parent atom. The H111—O11—H112 angle of the free water molecule was restrained to 109.47 (10)°.

### Crystal data

**Table:**

TbHP_2_O_7_·4H_2_O	*F*_000_ = 768
*M**_r_* = 405.9	*D*_x_ = 3.037 Mg m^−^^3^
Monoclinic, *P*2_1_/*n*	Mo *K*α radiation λ = 0.71069 Å
Hall symbol: -P 2yn	Cell parameters from 972 reflections
*a* = 6.6006 (6) Å	θ = 2.5–26.5º
*b* = 11.4744 (9) Å	µ = 8.38 mm^−^^1^
*c* = 11.7252 (13) Å	*T* = 295 K
β = 92.150 (8)º	Prism, colorless
*V* = 887.42 (15) Å^3^	0.14 × 0.06 × 0.03 mm
*Z* = 4	

### Data collection

**Table:**

Oxford Diffraction CCD diffractometer	1850 independent reflections
Radiation source: X-ray tube	1628 reflections with *I* > 3σ(*I*)
Monochromator: graphite	*R*_int_ = 0.019
Detector resolution: 8.3438 pixels mm^-1^	θ_max_ = 26.6º
*T* = 295 K	θ_min_ = 2.5º
ω scans	*h* = −8→7
Absorption correction: analytical[CrysAlis RED (Oxford Diffraction, 2005), using a multifaceted crystal model based on expressions derived by Clark & Reid (1995)]	*k* = −14→14
*T*_min_ = 0.329, *T*_max_ = 0.635	*l* = −14→14
8671 measured reflections	

### Refinement

**Table:**

Refinement on *F*^2^	H atoms treated by a mixture of independent and constrained refinement
*R*[*F*^2^ > 2σ(*F*^2^)] = 0.011	Weighting scheme based on measured s.u.'s *w* = 1/[σ^2^(*I*) + 0.0004*I*^2^]
*wR*(*F*^2^) = 0.034	(Δ/σ)_max_ = 0.009
*S* = 1.21	Δρ_max_ = 0.29 e Å^−^^3^
1850 reflections	Δρ_min_ = −0.21 e Å^−^^3^
155 parameters	Extinction correction: B-C type 1 Lorentzian isotropic [Becker, P. J. & Coppens, P. (1974). Acta Cryst. A30, 129–147]
10 restraints	Extinction coefficient: 2.9 (8)
9 constraints	

### Special details

**Table:**

Refinement. The refinement was carried out against all reflections. The conventional *R*-factor is always based on *F*. The goodness of fit as well as the weighted *R*-factor are based on *F* and *F*^2^ for refinement carried out on *F* and *F*^2^, respectively. The threshold expression is used only for calculating *R*-factors *etc*. and it is not relevant to the choice of reflections for refinement.The program used for refinement, Jana2000, uses the weighting scheme based on the experimental expectations, see _refine_ls_weighting_details, that does not force *S* to be one. Therefore the values of *S* may be larger then the ones from the *SHELX* program.

### Fractional atomic coordinates and isotropic or equivalent isotropic displacement parameters (Å^2^)

**Table:**

	*x*	*y*	*z*	*U*_iso_*/*U*_eq_	
Tb1	0.755557 (17)	0.417027 (10)	0.836213 (10)	0.00831 (5)	
P1	0.25551 (9)	0.54805 (6)	0.85787 (5)	0.00834 (18)	
P2	0.47954 (9)	0.63300 (5)	0.66266 (5)	0.00860 (18)	
O1	0.4213 (3)	0.45787 (15)	0.87403 (15)	0.0122 (5)	
O2	0.0629 (3)	0.50007 (16)	0.80317 (16)	0.0157 (5)	
O3	0.2158 (3)	0.61576 (15)	0.96588 (15)	0.0149 (5)	
O4	0.3423 (3)	0.57670 (15)	0.56618 (17)	0.0169 (6)	
O5	0.6553 (3)	0.55618 (15)	0.69557 (16)	0.0137 (5)	
O6	0.5259 (2)	0.75465 (15)	0.62791 (15)	0.0135 (5)	
O7	0.3378 (3)	0.64547 (14)	0.77094 (14)	0.0112 (5)	
O8	0.7671 (3)	0.62083 (18)	0.93419 (17)	0.0177 (6)	
O9	0.8201 (4)	0.34050 (17)	0.64753 (17)	0.0252 (7)	
O10	0.5535 (3)	0.24098 (19)	0.8262 (2)	0.0333 (7)	
O11	0.2577 (4)	0.36300 (19)	0.5980 (2)	0.0394 (9)	
H81	0.697 (4)	0.614 (3)	0.9904 (18)	0.0212*	
H111	0.294 (4)	0.3021 (18)	0.572 (3)	0.0472*	
H82	0.881 (2)	0.643 (3)	0.955 (2)	0.0212*	
H112	0.141 (2)	0.375 (3)	0.577 (3)	0.0472*	
H101	0.593 (5)	0.1757 (15)	0.810 (3)	0.04*	
H91	0.776 (5)	0.380 (3)	0.593 (2)	0.0303*	
H92	0.777 (4)	0.2742 (13)	0.633 (3)	0.0303*	
H41	0.323 (4)	0.5072 (10)	0.574 (3)	0.0203*	
H102	0.434 (2)	0.243 (3)	0.807 (3)	0.04*	

### Atomic displacement parameters (Å^2^)

**Table:**

	*U*^11^	*U*^22^	*U*^33^	*U*^12^	*U*^13^	*U*^23^
Tb1	0.00694 (8)	0.00827 (8)	0.00975 (8)	0.00064 (4)	0.00087 (5)	0.00041 (4)
P1	0.0067 (3)	0.0093 (3)	0.0092 (3)	0.0008 (2)	0.0021 (2)	0.0011 (2)
P2	0.0082 (3)	0.0085 (3)	0.0092 (3)	−0.0007 (2)	0.0009 (2)	0.0011 (2)
O1	0.0097 (9)	0.0127 (8)	0.0146 (9)	0.0029 (7)	0.0037 (7)	0.0037 (7)
O2	0.0095 (9)	0.0182 (9)	0.0192 (10)	−0.0040 (7)	−0.0004 (8)	0.0001 (7)
O3	0.0201 (10)	0.0137 (8)	0.0110 (10)	0.0048 (8)	0.0034 (7)	−0.0010 (7)
O4	0.0225 (11)	0.0120 (9)	0.0157 (10)	−0.0038 (8)	−0.0041 (8)	−0.0012 (7)
O5	0.0113 (9)	0.0156 (9)	0.0143 (10)	0.0031 (7)	0.0031 (8)	0.0039 (7)
O6	0.0141 (9)	0.0107 (8)	0.0159 (9)	−0.0034 (7)	0.0016 (7)	0.0022 (7)
O7	0.0115 (9)	0.0091 (8)	0.0133 (9)	0.0003 (7)	0.0055 (7)	0.0018 (7)
O8	0.0163 (11)	0.0208 (10)	0.0160 (11)	−0.0033 (9)	0.0013 (8)	0.0035 (8)
O9	0.0432 (13)	0.0160 (10)	0.0159 (11)	0.0057 (10)	−0.0061 (9)	−0.0017 (8)
O10	0.0138 (10)	0.0163 (10)	0.0697 (17)	−0.0019 (9)	−0.0005 (11)	−0.0122 (11)
O11	0.0541 (17)	0.0120 (11)	0.0542 (16)	−0.0102 (10)	0.0317 (14)	−0.0094 (10)

### Geometric parameters (Å, °)

**Table:**

Tb1—O1	2.3145 (17)	P1—O1	1.5129 (18)
Tb1—O2^i^	2.2877 (17)	P1—O2	1.5063 (18)
Tb1—O3^ii^	2.3514 (18)	P1—O3	1.5169 (19)
Tb1—O5	2.3718 (18)	P1—O7	1.6201 (18)
Tb1—O6^iii^	2.3842 (17)	P2—O4	1.562 (2)
Tb1—O8	2.605 (2)	P2—O5	1.4957 (19)
Tb1—O9	2.433 (2)	P2—O6	1.4891 (18)
Tb1—O10	2.421 (2)	P2—O7	1.6116 (18)
			
O1—Tb1—O2^i^	143.70 (6)	O6^iii^—Tb1—O8	128.03 (6)
O1—Tb1—O3^ii^	83.41 (6)	O6^iii^—Tb1—O9	75.70 (7)
O1—Tb1—O5	75.74 (6)	O6^iii^—Tb1—O10	71.65 (6)
O1—Tb1—O6^iii^	134.37 (6)	O8—Tb1—O9	136.20 (6)
O1—Tb1—O8	75.29 (6)	O8—Tb1—O10	141.01 (7)
O1—Tb1—O9	116.61 (7)	O9—Tb1—O10	76.69 (8)
O1—Tb1—O10	69.56 (7)	O1—P1—O2	113.46 (10)
O2^i^—Tb1—O3^ii^	101.23 (6)	O1—P1—O3	113.12 (10)
O2^i^—Tb1—O5	80.10 (6)	O1—P1—O7	107.03 (10)
O2^i^—Tb1—O6^iii^	79.67 (6)	O2—P1—O3	111.92 (11)
O2^i^—Tb1—O8	71.90 (6)	O2—P1—O7	106.35 (10)
O2^i^—Tb1—O9	79.03 (7)	O3—P1—O7	104.14 (10)
O2^i^—Tb1—O10	146.08 (7)	O4—P2—O5	111.48 (10)
O3^ii^—Tb1—O5	143.50 (6)	O4—P2—O6	107.99 (10)
O3^ii^—Tb1—O6^iii^	71.04 (6)	O4—P2—O7	105.60 (10)
O3^ii^—Tb1—O8	73.05 (6)	O5—P2—O6	117.25 (10)
O3^ii^—Tb1—O9	146.04 (7)	O5—P2—O7	108.46 (10)
O3^ii^—Tb1—O10	86.44 (8)	O6—P2—O7	105.28 (10)
O5—Tb1—O6^iii^	143.13 (6)	H81—O8—H82	109 (3)
O5—Tb1—O8	72.85 (6)	H91—O9—H92	104 (3)
O5—Tb1—O9	70.39 (6)	H101—O10—H102	106 (3)
O5—Tb1—O10	112.90 (7)	H111—O11—H112	109 (3)

Symmetry codes: (i) *x*+1, *y*, *z*; (ii) −*x*+1, −*y*+1, −*z*+2; (iii) −*x*+3/2, *y*−1/2, −*z*+3/2.

### Hydrogen-bond geometry (Å, °)

**Table:**

*D*—H···*A*	*D*—H	H···*A*	*D*···*A*	*D*—H···*A*
O4—H41···O11	0.813 (13)	1.736 (14)	2.546 (3)	174 (3)
O8—H81···O1^ii^	0.82 (2)	1.98 (3)	2.762 (3)	159 (3)
O8—H82···O3^i^	0.822 (18)	2.231 (14)	2.972 (3)	150 (3)
O9—H91···O4^iv^	0.83 (3)	2.06 (3)	2.851 (3)	161 (3)
O9—H92···O8^iii^	0.828 (18)	1.95 (2)	2.750 (3)	164 (3)
O10—H101···O5^iii^	0.82 (2)	2.16 (3)	2.880 (3)	147 (3)
O10—H102···O7^v^	0.812 (16)	2.28 (2)	2.991 (3)	147 (3)
O11—H111···O3^v^	0.80 (2)	2.18 (2)	2.941 (3)	157 (3)

Symmetry codes: (ii) −*x*+1, −*y*+1, −*z*+2; (i) *x*+1, *y*, *z*; (iv) −*x*+1, −*y*+1, −*z*+1; (iii) −*x*+3/2, *y*−1/2, −*z*+3/2; (v) −*x*+1/2, *y*−1/2, −*z*+3/2.
